# Early- versus late-onset Alzheimer’s disease in clinical practice: cognitive and global outcomes over 3 years

**DOI:** 10.1186/s13195-017-0294-2

**Published:** 2017-08-31

**Authors:** Carina Wattmo, Åsa K. Wallin

**Affiliations:** Clinical Memory Research Unit, Department of Clinical Sciences, Malmö, Lund University, SE-205 02 Malmö, Sweden

**Keywords:** Cognition, Cholinesterase inhibitors, Early-onset Alzheimer’s disease, Late-onset Alzheimer’s disease, Predictors, Longitudinal study, Mixed-effects models

## Abstract

**Background:**

Whether age at onset influences Alzheimer’s disease (AD) progression and the effectiveness of cholinesterase inhibitor (ChEI) therapy is not clear. We aimed to compare longitudinal cognitive and global outcomes in ChEI-treated patients with early-onset Alzheimer’s disease (EOAD) versus late-onset Alzheimer’s disease (LOAD) in clinical practice.

**Methods:**

This 3-year, prospective, observational, multicentre study included 1017 participants with mild to moderate AD; 143 had EOAD (age at onset < 65 years) and 874 had LOAD (age at onset ≥ 65 years). At baseline and semi-annually, patients were assessed using cognitive, global and activities of daily living (ADL) scales, and the dose of ChEI was recorded. Potential predictors of decline were analysed using mixed-effects models.

**Results:**

Six-month response to ChEI therapy and long-term prognosis in cognitive and global performance were similar between the age-at-onset groups. However, deterioration was significantly faster when using the Alzheimer’s Disease Assessment Scale–Cognitive subscale (ADAS-Cog) over 3 years in participants with EOAD than in those with LOAD; hence, prediction models for the mean ADAS-Cog trajectories are presented. The younger cohort had a larger proportion of homozygote apolipoprotein E (APOE) ε4 allele carriers than the older cohort; however, APOE genotype was not a significant predictor of cognitive impairment in the multivariate models. A slower rate of cognitive progression was related to initiation of ChEIs at an earlier stage of AD, higher ChEI dose and fewer years of education in both groups. In LOAD, male sex, better instrumental ADL ability and no antipsychotic drug use were additional protective characteristics. The older patients received a lower ChEI dose than the younger individuals during most of the study period.

**Conclusions:**

Although the participants with EOAD showed a faster decline in ADAS-Cog, had a longer duration of AD before diagnosis, and had a higher frequency of two APOE ε4 alleles than those with LOAD, the cognitive and global responses to ChEI treatment and the longitudinal outcomes after 3 years were similar between the age-at-onset groups. A higher mean dose of ChEI and better cognitive status at the start of therapy were independent protective factors in both groups, stressing the importance of early treatment in adequate doses for all patients with AD.

## Background

People who have a clinical onset of Alzheimer’s disease (AD) before the age of 65 years are diagnosed with early-onset Alzheimer’s disease (EOAD). The prevalence of patients with EOAD is low, but it varies in studies from 6% to 16% [1–3]. Some observations suggest that EOAD might be a separate, more severe entity than late-onset Alzheimer’s disease (LOAD). Researchers in neuropathological studies have reported that younger patients with AD exhibited higher burdens of neuritic plaques and neurofibrillary tangles, as well as greater synapse loss, than older individuals [4]. Moreover, patients with AD who died before 80 years of age had a more widespread and severe cholinergic deficit with abnormalities in other neurotransmitters (e.g., noradrenaline) compared with those who died at older ages [5]. Regarding cognition, the patients with EOAD demonstrated more impairment in language and concentration, whereas the LOAD cohort showed difficulties in memory and orientation [6]. Clinical diagnosis of AD is often missed in individuals with early onset because of the atypical symptoms and non-amnestic presentations [7]. Younger persons are often more educated than older individuals and have a higher cognitive reserve capacity that could also lead to a delayed diagnosis [8]. Therefore, antidementia therapy might be initiated in a later stage of the disease, which may impair the efficacy of treatment in EOAD.

Currently, the main therapy for mild to moderate AD is cholinesterase inhibitors (ChEIs). Positive cognitive and global symptomatic effects compared with placebo have been reported mainly in 6-month randomised clinical trials [9]. All these trials, as well as long-term extensions [10, 11] and observational studies of ChEI treatment in AD [12, 13], have enrolled participants regardless of age at onset. However, the level of short-term therapeutic response and longitudinal outcome may vary depending on age at onset of AD. By investigating the entire mild to moderate Swedish Alzheimer Treatment Study (SATS) cohort, our group found that both cognitive response to ChEIs and prognosis after 3 years were better in older patients with AD than in younger individuals. In addition, we showed that male sex, absence of the apolipoprotein E (APOE) ε4 allele, use of non-steroidal anti-inflammatory drugs (NSAIDs)/acetylsalicylic acids, and receiving a higher ChEI dose, regardless of type of drug, were independent predictors related to slower cognitive deterioration [13]. The aforementioned association between age and cognitive performance was not observed in another study [14]. Conversely, in a 3-month donepezil study, participants younger than 66 years of age exhibited greater improvement than older patients [15]. No longer-term studies have previously reported possible socio-demographic and clinical characteristics or aspects of ChEI therapy (e.g., drug agent and dose) that might affect the cognitive trajectory in a cohort with exclusively EOAD.

A faster rate of cognitive progression among younger individuals with AD was shown in some studies [16, 17], whereas others demonstrated a similar decline between the age groups [18, 19] or more rapid impairment in older participants [20]. These mixed and conflicting observations may depend on differences in sample size and follow-up period, as well as possible confounding factors such as APOE genotype, education level, activities of daily living (ADL) capacity, and concomitant disorders, which were not considered by most earlier studies on EOAD versus LOAD [21]. The SATS includes multiple variables (e.g., the above-mentioned covariates) that have not been evaluated simultaneously in prior publications. By dividing the SATS cohort into subsets according to age at onset and separately analysing these groups with an advanced multivariate statistical approach (mixed-effects models), a better understanding of the course of AD and potential predictive characteristics in younger versus older persons with AD might be expected.

The aims of this study were (1) to describe and compare cognitive and global longitudinal outcomes between ChEI-treated patients with EOAD and LOAD in clinical practice and (2) to identify socio-demographic and clinical factors (e.g., sex, APOE genotype, years of education, concomitant medications) and aspects of ChEI treatment (drug agent, dose) that could affect the cognitive abilities in the respective groups.

## Methods

### Study and participants

The SATS is a 3-year, prospective, open-label, non-randomised, multicentre study that was started to investigate the long-term effectiveness of ChEI therapy (donepezil, rivastigmine and galantamine) in clinical practice. Different findings from the SATS have been presented in several publications (e.g., [12, 13, 22–24]). In total, 1258 participants with AD were recruited from 14 memory clinics located in different geographical areas across Sweden. All 1021 patients exhibiting a baseline Mini Mental State Examination (MMSE) [25] score ranging from 10 to 26 and an age at onset of AD (4 participants had missing data) were included in the current study. Of these, 143 individuals were defined as having EOAD (age at onset of AD < 65 years), and 874 were defined as having LOAD (age at onset of AD ≥ 65 years); hence, 1017 participants were enrolled.

Considered for inclusion in the SATS were outpatients aged ≥ 40 years who met the criteria for the clinical diagnosis of dementia, as defined in the *Diagnostic and Statistical Manual of Mental Disorders, Fourth Edition* [26], and for possible or probable AD according to the criteria of the National Institute of Neurological and Communicative Disorders and Stroke and the Alzheimer’s Disease and Related Disorders Association [27]. All patients were diagnosed by physicians who specialise in dementia disorders. The dementia specialist estimated the age at onset on the basis of an interview with the caregiver (usually the spouse or an adult child) regarding observations of early symptoms of AD. Moreover, the selected individuals had to live at their own home at the time of AD diagnosis, to have a responsible caregiver and to be assessable with the MMSE at the start of the ChEI treatment (baseline). The exclusion criteria were not fulfilling the diagnostic criteria for AD, already receiving active ChEI therapy or having contra-indications to ChEIs.

After inclusion in the study and the baseline evaluations, the participants were prescribed ChEI treatment as part of the ordinary Swedish health-care system and in accordance with the approved product labelling. All patients started with donepezil 5 mg, rivastigmine 3 mg, or galantamine 8 mg, as in routine clinical practice. The SATS is an observational study, and the choice of drug type and all decisions regarding dosage were left entirely up to the dementia specialist’s discretion and professional judgement. Most individuals received an increased dose after 4–8 weeks of treatment, and we aimed at further dose increases depending on the chosen ChEI agent. However, for some participants, the dose was reduced because of side effects. The ChEI dose was recorded after 2 months of therapy and then every 6 months after baseline. Medications other than ChEIs were documented at baseline and allowed during the study, with the exception of memantine. If the patient stopped taking the ChEI or if memantine was initiated, the individual discontinued the SATS at that time point. The date of and reason for any drop-out from the SATS were recorded.

### Outcome measures

The SATS patients were investigated in a well-structured follow-up programme in which researchers evaluated cognitive, global and ADL performance at the start of ChEI treatment, after 2 months (MMSE and global rating only) and semi-annually over 3 years. Cognitive status was assessed using the MMSE, with scores ranging from 0 to 30 (a lower score indicating more impaired cognition), and the Alzheimer’s Disease Assessment Scale–Cognitive subscale (ADAS-Cog) [28], with a total range of 0 to 70 (a higher score indicating more impaired cognition). The Clinician Interview-Based Impression of Change (CIBIC) [29] was used as a global rating of ‘change from the initiation of ChEI therapy’. The evaluations were performed at all intervals using a 7-point scale ranging from 1 (very much improved) to 7 (marked worsening). Three groups of response were defined at each CIBIC interval: 1–3 indicated improvement, 4 indicated no change and 5–7 indicated worsening. No guidelines or descriptors were provided to define the individual ratings. The classification between, for example, minimally improved or very much improved was left to the dementia specialist’s clinical judgement.

The Instrumental Activities of Daily Living (IADL) scale [30] consists of eight different items: ability to use the telephone, shopping, food preparation, housekeeping, laundry, mode of transportation, responsibility for one’s own medications and ability to handle finances. Each item was scored from 1 (no impairment) to 3–5 (severe impairment), which yielded a total range of 8–31 points. The Physical Self-Maintenance Scale [30] consists of six different items: toileting, feeding, dressing, grooming, physical ambulation and bathing. Each item was scored from 1 (no impairment) to 5 (severe impairment), which allowed a total range of 6–30 points. Trained dementia nurses assessed the ADL performance on the basis of interviews with the caregiver. To facilitate the comparison of rates in MMSE and ADAS-Cog scores, changes in score were converted to positive values, which were indicative of improvement, and negative values, which were indicative of deterioration.

### Statistical analyses

IBM SPSS Statistics for Windows version 22.0 software (IBM Corporation, Armonk, NY, USA) was used to perform the statistical analyses. The level of significance was defined as *p* < 0.05 if not otherwise specified, and all tests were two-tailed. Observed-case analyses were used to avoid overestimation of the treatment effect by imputing higher previous outcome scores in a longitudinal study of a progressively deteriorating disease. Parametric tests were used because of the large sample size and the approximately normally distributed continuous potential predictors. Independent-sample *t* tests were used to compare the differences between the means obtained for two groups, such as EOAD and LOAD, and χ^2^ tests were used to analyse categorical variables.

Mixed, linear and non-linear fixed and random coefficient regression models using the participant as a hierarchical variable (i.e., to allow intra-individual correlation) were performed. The mixed-effects models method also takes into account the varying number of evaluations available for each patient and unequal time intervals between the follow-up visits, which are common statistical limitations found in long-term studies [31]. The non-completers contributed information during the time of participation; hence, we considered the trajectories of all SATS patients.

The dependent variables were the cognitive scores assigned at the second and subsequent visits for each individual; that is, the mixed-effects models do not intend to predict the scores at the initiation of ChEI therapy. The following described independent variables were included in the models. First, the initial cognitive scores for each participant (to adjust for baseline differences) and their interaction with linear and quadratic terms for time in the study (to enable a non-linear rate of change in the models) were included as fixed effects; that is, time in months (and time in months^2^) × MMSE (or ADAS-Cog) baseline score. Time was defined as the exact number of months between the start of ChEI treatment and each visit, thus using all data points at the actual time intervals. Secondly, several possible socio-demographic and clinical predictors were included as fixed effects in the models, such as sex; age at the start of ChEI therapy; clinician’s estimated duration of AD; years of education; presence of the APOE ε4 allele (no/yes); solitary living (no/yes); IADL and basic ADL capacity; number of medications at baseline; and specific concomitant medications (no/yes for each group), including antihypertensive/cardiac therapy, antidiabetics, asthma medications, thyroid therapy, lipid-lowering agents, oestrogens, NSAIDs/acetylsalicylic acid, antidepressants, antipsychotics and anxiolytics/sedatives/hypnotics. Thirdly, the effect of the different ChEI agents was analysed using the type of drug (coded as a set of dummy variables) and dosages. The terms ‘ChEI agent × dose’ and ‘age × ChEI dose’ were also included in the models. The ChEI dose could vary during the treatment period for an individual patient and between patients; therefore, the mean dose used during the entire follow-up period was calculated for each participant. In cases of drop-out, the mean dose used during the individual’s time of participation in the SATS was calculated. To obtain a similar metric for percentage maximum dosage for each of the three ChEIs, the mean dose was divided by the maximum recommended dose for each drug, namely 10 mg for donepezil, 12 mg for rivastigmine (oral administration) and 24 mg for galantamine. Lastly, some potential interactions (sex, age or education) with cognitive severity at baseline or with time in the study were included in the models. The random terms were an intercept and time in months, with a variance components covariance matrix. Non-significant variables (*p* > 0.05) were eliminated in a backward stepwise manner. The hierarchical principle was applied in the mixed-effects models; variables that appeared in significant interactions were not considered for elimination.

## Results

### Socio-demographic and clinical characteristics according to age at onset of AD

The 1017 SATS participants were divided into two cohorts according to their age at onset of AD: EOAD (< 65 years, *n* = 143 [14%]) and LOAD (≥ 65 years, *n* = 874 [86%]). The socio-demographic and clinical characteristics of the two cohorts are presented in Table 1. The presence of two APOE ε4 alleles was more frequent, and the proportion of one or no ε4 alleles was less frequent, among the patients with EOAD than in those with LOAD [χ^2^(2) = 23.98, *p <* 0.001]. The mean duration of illness was longer [*t*_(1015)_ = 4.36, *p* < 0.001], and the level of education higher [*t*_(1015)_ = 2.93, *p* = 0.004], among the younger than the older individuals. Cognitive ability at the initiation of ChEI therapy did not differ between the onset groups. Among the participants with EOAD, a lower percentage received donepezil and a higher percentage received rivastigmine or galantamine [χ^2^(2) = 8.09, *p* = 0.017]. The mean dose of donepezil during the study was higher in the younger cohort [*t*_(514)_ = 2.32, *p* = 0.020], but it was similar between the groups for the other two ChEI agents.

**Table 1:** Socio-demographic and clinical characteristics of the SATS participants (*n* = 1017)

Variable	EOAD (*n* = 143 [14%])	LOAD (*n* = 874 [86%])	*p* Value
	*n*/%	*n*/%	
Female sex	82/57%	568/65%	0.091
APOE genotype (*n* = 996)			< 0.001
No ε4 alleles	36/25%	284/33%	
One ε4 allele	66/46%	459/54%	
Two ε4 alleles	41/29%	110/13%	
Solitary living at baseline	30/21%	322/37%	< 0.001
Completion rate after 3 years	57/40%	318/36%	0.425
Antihypertensives/cardiac therapy	28/20%	384/44%	< 0.001
Antidiabetics	5/3%	45/5%	0.397
Asthma medication	9/6%	34/4%	0.185
Thyroid therapy	9/6%	76/9%	0.336
Lipid-lowering agents	16/11%	101/12%	0.898
Oestrogens	9/6%	60/7%	0.801
NSAIDs/acetylsalicylic acid	15/10%	288/33%	< 0.001
Antidepressants	41/29%	215/25%	0.298
Antipsychotics	2/1%	43/5%	0.058
Anxiolytics/sedatives/hypnotics	6/4%	141/16%	< 0.001
Variable	Mean ± SD	Mean ± SD	*p* Value
Estimated age at onset, years, range	58.6 ± 4.7, 45–64	74.4 ± 4.9, 65–88	< 0.001
Estimated duration of AD at baseline, years	4.1 ± 3.4	2.9 ± 1.7	< 0.001
Age at first assessment, years	62.7 ± 5.4	77.3 ± 4.7	< 0.001
Education, years	10.1 ± 2.8	9.3 ± 2.5	0.004
MMSE score at baseline	21.4 ± 3.8	21.4 ± 3.7	0.987
ADAS-Cog score (0–70) at baseline	19.5 ± 9.6	21.0 ± 8.8	0.074
IADL score at baseline	13.9 ± 5.3	16.3 ± 5.4	< 0.001
PSMS score at baseline	6.7 ± 1.2	7.6 ± 2.4	< 0.001
Number of concomitant medications at baseline	1.8 ± 1.7	3.1 ± 2.5	< 0.001
Mean dose of ChEI during follow-up period, mg			
Donepezil (*n* = 516)^a^	7.4 ± 1.9 (40%)	6.8 ± 1.7 (52%)	0.020
Rivastigmine (*n* = 211)^a^	6.6 ± 2.3 (26.5%)	6.0 ± 2.1 (20%)	0.100
Galantamine (*n* = 290)^a^	15.8 ± 3.6 (33.5%)	15.1 ± 3.8 (28%)	0.184

*Abbreviations: AD* Alzheimer’s disease, *ADAS-Cog* Alzheimer’s Disease Assessment Scale–Cognitive subscale, *APOE* Apolipoprotein E, *ChEI* Cholinesterase inhibitor, *EOAD* Early-onset Alzheimer’s disease, *IADL* Instrumental Activities of Daily Living scale, *LOAD* Late-onset Alzheimer’s disease, *MMSE* Mini Mental State Examination, *NSAIDs* Non-steroidal anti-inflammatory drugs, *PSMS* Physical Self-Maintenance Scale, *SATS* Swedish Alzheimer Treatment Study, *SD* Standard deviation

^a^Percentage of patients in each group who received the specific ChEI agent in parentheses (*p* = 0.017 by χ^2^ test)

### Comparison of longitudinal outcomes between EOAD versus LOAD

Regarding the MMSE score, 71% of the patients with EOAD and 64% of those with LOAD showed improvement/no change (≥ 0-point change) after 6 months of ChEI treatment [χ^2^(1) = 2.60, *p* = 0.107]. Improvement/no change (≥ 0-point change) in ADAS-Cog score was observed for 50% of the younger and 56% of the older cohort after 6 months [χ^2^(1) = 1.60, *p* = 0.206].

**Fig 1:**
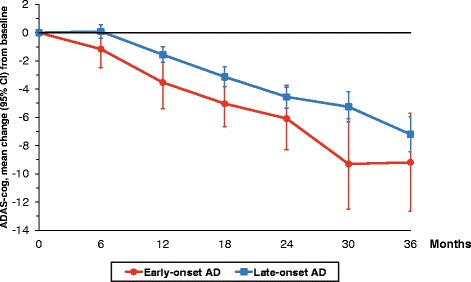
Cognitive outcome over 3 years of cholinesterase inhibitor (ChEI) treatment. The mean changes in Alzheimer’s Disease Assessment Scale–Cognitive subscale (ADAS-Cog) score with 95% CI from the start of ChEI therapy (baseline) over 3 years, by age at onset of Alzheimer’s disease (AD). The patients with early-onset Alzheimer’s disease showed a more rapid rate of cognitive decline from baseline after 12 months (*p* = 0.045), 18 months (*p* = 0.035) and 30 months (*p* = 0.019) of treatment

The mean (95% CI) MMSE and ADAS-Cog scores and the changes from baseline scores over the 3-year study by age-at-onset group are shown in Table 2. Using the ADAS-Cog scale, the rate of cognitive decline was faster among the EOAD participants at 12, 18 and 30 months after the initiation of ChEI therapy (Fig. 1). The mean (95% CI) semi-annual rates of change in the ADAS-Cog score at each time point for the younger and older cohorts, respectively, were, for 6–12 months, –2.4 (–3.8, –1.0) versus –1.9 (–2.4, –1.4) points (*p* = 0.483); for 12–18 months, –2.9 (–4.0, –1.8) versus –2.0 (–2.6, –1.5) points (*p* = 0.207); for 18–24 months, –1.5 (–2.9, –0.1) versus –1.9 (–2.5, –1.3) points (*p* = 0.643); for 24–30 months, –2.9 (–4.7, –1.2) versus –2.1 (–2.7, –1.5) points (*p* = 0.325); and for 30–36 months, –2.6 (–4.6, –0.6) versus –2.7 (–3.5, –1.8) points (*p* = 0.945). No significant difference in disease progression over time between the onset groups was detected when the MMSE score was used.

**Table 2:** Changes in cognitive and global performance, and dose of cholinesterase inhibitors, during 3 years of therapy, by age-at-onset group

Number of patients with EOAD/LOAD	MMSE score/MMSE change from baseline^a^			ADAS-Cog score (0–70)/ADAS-Cog change from baseline^a^			CIBIC score, % of improved or unchanged remaining patients			ChEI dose^a,b^		
	EOAD	LOAD	*p* Value	EOAD	LOAD	*p* Value	EOAD	LOAD	*p* Value	EOAD	LOAD	*p* Value
2 months (*n* = 138/827)	22.5 (21.7, 23.3)/1.1 (0.7, 1.6)	22.3 (22.1, 22.6)/0.9 (0.7, 1.1)	0.674/0.313	NA	NA	NA	90	93	0.261	63 (60, 67)	61 (59, 62)	0.160
6 months (*n* = 126/752)	21.6 (20.7, 22.5)/0.4 (−0.2, 0.9)	21.8 (21.5, 22.1)/0.4 (0.2, 0.7)	0.671/0.829	20.7 (18.8, 22.6)/−1.2 (−2.5, 0.2)	20.4 (19.7, 21.1)/0.1 (−0.4, 0.6)	0.747/0.055	76	76	0.978	71 (67, 75)	68 (67, 70)	0.233
12 months (*n* = 111/669)	20.2 (19.0, 21.4)/−1.0 (−1.8, −0.2)	21.1 (20.7, 21.5)/−0.5 (−0.8, −0.2)	0.169/0.218	23.3 (20.7, 25.9)/−3.5 (−5.4, −1.7)	21.5 (20.7, 22.3)/−1.6 (−2.1, −1.0)	0.199/0.045	49	57	0.116	80 (76, 84)	73 (71, 75)	0.005
18 months (*n* = 101/530)	20.1 (18.9, 21.3)/−1.5 (−2.3, −0.7)	20.4 (19.9, 20.8)/−1.4 (−1.7, −1.0)	0.692/0.727	24.5 (21.7, 27.2)/−5.0 (−6.7, −3.4)	22.7 (21.7, 23.8)/−3.1 (−3.8, −2.4)	0.235/0.035	44	50	0.265	83 (79, 88)	77 (75, 79)	0.010
24 months (*n* = 84/480)	18.5 (16.9, 20.2)/−3.0 (−4.3, −1.7)	19.8 (19.3, 20.3)/−2.2 (−2.6, −1.8)	0.147/0.243	23.8 (20.3, 27.2)/−6.1 (−8.3, −3.9)	23.5 (22.3, 24.7)/−4.5 (−5.4, −3.7)	0.890/0.164	32	40	0.162	86 (82, 90)	78 (76, 80)	0.002
30 months (*n* = 66/346)	18.7 (16.7, 20.6)/−3.2 (−4.6, −1.7)	19.6 (19.0, 20.2)/−2.6 (−3.1, −2.1)	0.385/0.448	27.0 (22.7, 31.2)/−9.3 (−12.5, −6.1)	23.9 (22.5, 25.3)/−5.3 (−6.3, −4.2)	0.183/0.019	29	34	0.452	85 (80, 90)	78 (76, 81)	0.020
36 months (*n* = 57/318)	17.5 (15.4, 19.5)/−4.1 (−5.6, −2.6)	19.3 (18.6, 19.9)/−3.1 (−3.7, −2.6)	0.091/0.230	26.3 (21.4, 31.1)/−9.2 (−12.7, −5.7)	24.8 (23.2, 26.5)/−7.2 (−8.4, −6.0)	0.517/0.227	32	29	0.650	88 (83, 93)	80 (77, 82)	0.005

*ADAS-Cog* Alzheimer’s Disease Assessment Scale–Cognitive subscale, *ChEI* Cholinesterase inhibitor, *CIBIC* Clinician Interview-Based Impression of Change, *EOAD* Early-onset Alzheimer’s disease, *LOAD* Late-onset Alzheimer’s disease, *MMSE* Mini Mental State Examination, *NA* Not assessed

For clarity, clinical improvements for both MMSE and ADAS-Cog scales have been tabulated as positive changes from the start of ChEI treatment (baseline)

^a^Mean (95% CI)

^b^Mean percentage of the maximum recommended dose, namely 10 mg for donepezil, 12 mg for rivastigmine and 24 mg for galantamine

**Fig 2:**
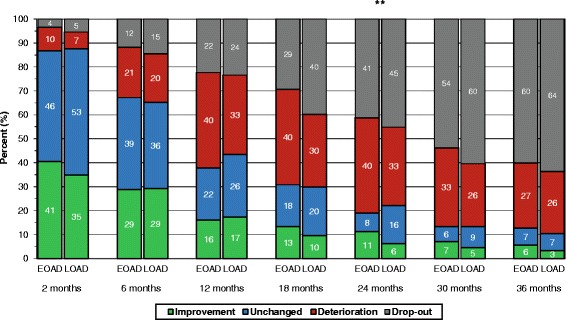
Global outcome over 3 years of cholinesterase inhibitor (ChEI) treatment. The proportion of patients is shown according to differences in treatment response in global performance (Clinician Interview-Based Impression of Change (CIBIC)) from the start of ChEI therapy over 3 years for early-onset Alzheimer’s disease (EOAD) versus late-onset Alzheimer’s disease (LOAD). No significant differences in global response were observed between the two groups, except after 24 months (*p* = 0.005) of treatment. CIBIC scores 1–3 were considered as improvement, 4 as unchanged, and 5–7 as deterioration

The percentages of the EOAD versus LOAD cohorts according to changes in global performance (CIBIC) after 2 months and semi-annually over 3 years from the start of ChEIs are illustrated in Fig. 2. The proportions of the remaining younger and older patients who exhibited improvement or no change in CIBIC score at each visit are presented in Table 2; however, no differences between the two groups were found. The individuals with EOAD and LOAD, respectively, were further divided into APOE genotypes. No significant differences in changes in cognitive or global capacities after 3 years of ChEI treatment were observed between these groups. The mean (95% CI) percentage of maximum ChEI dose was higher in EOAD than in LOAD participants at all visits after 12 months of therapy (Table 2).

In total, 86 patients (60%) with EOAD and 556 (64%) with LOAD did not complete the 3-year SATS [χ^2^(1) = 0.64, *p* = 0.425]. The reasons for drop-out in this cohort have been reported previously [24]. In the EOAD group, the completers received a greater percentage (mean ± SD) of the maximum recommended ChEI dose during the study [76 ± 14% versus 60 ± 19%, *t*_(141)_ = 5.66, *p* < 0.001]. No significant differences between the younger completers and drop-outs were found regarding cognitive or global status at the start of treatment. In the LOAD group, the completers also received a greater mean percentage of the maximum recommended ChEI dose during the study [69 ± 18% versus 59 ± 18%, *t*_(872)_ = 8.01, *p* < 0.001]. The older completers exhibited significantly better cognitive [mean ± SD, MMSE score 22.4 ± 3.4 versus 20.9 ± 3.8 points, *t*_(872)_ = 6.06, *p* < 0.001; ADAS-Cog score 18.6 ± 8.4 versus 22.4 ± 8.7 points, *t*_(858)_ = −6.30, *p* < 0.001] and global (CIBIC median score 3 versus 4 points, median test, *p* = 0.017) performance at baseline than the drop-outs. In addition, antipsychotic use was less frequent among the completers with LOAD [6 (2%) versus 37 (7%) patients, χ^2^(1) = 9.83, *p* = 0.002]. The other variables of interest in this study, such as sex, APOE genotype, age at baseline, duration of AD, years of education, number of concomitant medications and other specific medications received, did not differ between the completers and those who discontinued the study in any of the age-at-onset groups. Fifteen (10%) of the younger and 66 (8%) of the older individuals dropped out because of initiation of concomitant memantine therapy [χ^2^(1) = 1.45, *p* = 0.229]; their mean length (95% CI) of participation in the study was 23.1 (20.1–26.2) versus 19.7 (17.8–21.5) months [*t*_(79)_ = 1.66, *p* = 0.101], respectively.

### Predictors of disease progression in the respective age-at-onset groups

In the mixed-effects models, only participants with three or more assessments were included to enable analyses of a non-linear rate of cognitive change (EOAD *n* = 128 [90%]; LOAD *n* = 774 [89%]). The models were performed to identify the socio-demographic and clinical predictors that affected the patients’ longitudinal trajectories (EOAD 667 data points, LOAD 3733 data points). The percentages of variance that accounted for the dependent variable regarding all fixed factors were 54.7% for MMSE and 53.6% for ADAS-Cog in the EOAD group and 51.1% for MMSE and 55.3% for ADAS-Cog in the LOAD group. This indicates a good fit of the models (*p* < 0.001 for all models). The mixed-effects models, significant predictors and unstandardised β coefficients with 95% CIs are presented in Tables 3 and 4.

**Table 3:** Factors affecting long-term outcome with Mini Mental State Examination score as dependent variable, by age-at-onset group

	EOAD			LOAD		
Percentage of variance accounted for, all fixed terms	54.7%, *p* < 0.001			51.1%, *p* < 0.001		
Significant predictors in final mixed models	β Value	95% CI	*p* Value	β Value	95% CI	*p* Value
Fixed terms						
Intercept	2.512	0.103, 4.922	0.041	−36.137	−52.439, −19.835	< 0.001
Time in months from baseline	−0.487	−0.743, −0.230	< 0.001	−0.508	−0.609, −0.407	< 0.001
MMSE score at baseline	0.942	0.830, 1.054	< 0.001	3.051	2.201, 3.901	<0.001
MMSE score at baseline^2^			NS	−0.019	−0.029, −0.008	0.001
Time in months × MMSE score at baseline	0.017	0.005, 0.029	0.006	0.021	0.017, 0.026	< 0.001
Time in months^2^ × MMSE score at baseline	−0.0001	−0.0002, −0.0001	< 0.001	−0.00005	−0.00008, −0.00002	0.001
Background variables						
Sex (male = 0, female = 1)			NS	−0.375	−0.727, −0.023	0.037
Antipsychotics (no = 0, yes = 1)			NS	−0.984	−1.808, −0.161	0.019
Age at first assessment, years			NS	0.481	0.279, 0.683	< 0.001
Age × MMSE score at baseline			NS	−0.021	−0.031, −0.012	< 0.001
Education, years			NS	0.084	0.010, 0.158	0.027
Time in months × education, years			NS	−0.011	−0.017, −0.004	0.001
IADL score at baseline			NS	−0.110	−0.147, −0.073	< 0.001
ChEI dose^a^			NS	0.011	0.002, 0.021	0.020
Random terms (variance)						
Intercept	2.617	1.516, 4.517	< 0.001	2.770	2.273, 3.376	< 0.001
Time in months	0.045	0.031, 0.067	< 0.001	0.025	0.021, 0.029	< 0.001

*Abbreviations: AD* Alzheimer’s disease, *ChEI* Cholinesterase inhibitor, *EOAD* Early-onset Alzheimer’s disease, *IADL* Instrumental Activities of Daily Living Scale, *LOAD* Late-onset Alzheimer’s disease, *MMSE* Mini Mental State Examination, *NS* Not significant, *PSMS* Physical Self-Maintenance Scale

Number of apolipoprotein E ε4 alleles, solitary living, duration of AD, PSMS score at baseline, number of medications and the specific concomitant medications used at baseline except for antipsychotics, as well as the variable comparing the ChEI agents, were not significant predictors in the models. β Values were unstandardised and are expressed per 1-unit increase for continuous variables and for the condition present in dichotomous variables

^a^Mean percentage of the maximum recommended dose, namely 10 mg for donepezil, 12 mg for rivastigmine and 24 mg for galantamine

**Table 4:** Factors affecting long-term outcome with Alzheimer’s Disease Assessment Scale–Cognitive subscale as dependent variable, by age-at-onset group

	EOAD			LOAD		
Percentage of variance accounted for, all fixed terms	53.6%, *p* < 0.001			55.3%, *p* < 0.001		
Significant predictors in final mixed models	β Value	95% CI	*p* Value	β Value	95% CI	*p* Value
Fixed terms						
Intercept	16.592	7.605, 25.579	< 0.001	−28.833	−47.874, −9.793	0.003
Time in months from baseline	−0.352	−0.729, 0.026	0.067	−0.228	−0.397, −0.060	0.008
ADAS-Cog score at baseline	0.708	0.567, 0.849	< 0.001	2.688	1.838, 3.538	< 0.001
Time in months × ADAS-Cog score at baseline	0.022	0.013, 0.030	< 0.001	0.022	0.018, 0.026	< 0.001
Background variables						
Sex (male = 0, female = 1)			NS	−2.177	−4.525, 0.171	0.069
Sex × ADAS-Cog score at baseline			NS	0.150	0.043, 0.257	0.006
Age at first assessment, years			NS	0.422	0.175, 0.668	0.001
Age × ADAS-Cog score at baseline			NS	−0.026	−0.037, −0.015	< 0.001
Education, years	−0.569	−1.091, −0.047	0.033	−0.073	−0.275, 0.129	0.478
Time in months × education, years	0.041	0.009, 0.073	0.013	0.016	0.002, 0.030	0.029
IADL score at baseline			NS	0.273	0.175, 0.371	< 0.001
ChEI dose^a^	−0.097	−0.183, −0.011	0.027	−0.045	−0.070, −0.021	< 0.001
Random terms (variance)						
Intercept	32.457	21.150, 49.808	< 0.001	13.012	10.010, 16.915	< 0.001
Time in months	0.145	0.096, 0.221	< 0.001	0.103	0.086, 0.124	< 0.001

*Abbreviations: AD* Alzheimer’s disease, *ADAS-Cog* Alzheimer’s Disease Assessment Scale–Cognitive subscale, *ChEI* Cholinesterase inhibitor, *EOAD* Early-onset Alzheimer’s disease, *IADL* Instrumental Activities of Daily Living Scale, *LOAD* Late-onset Alzheimer’s disease, *NS* Not significant, *PSMS* Physical Self-Maintenance Scale

Number of apolipoprotein E ε4 alleles, solitary living, duration of AD, PSMS score at baseline, number of medications and the specific concomitant medications used at baseline, as well as the variable comparing the ChEI agents, were not significant predictors in the models. β Values were unstandardised and are expressed per 1-unit increase for continuous variables and for the condition present in dichotomous variables

^a^Mean percentage of the maximum recommended dose, namely 10 mg for donepezil, 12 mg for rivastigmine and 24 mg for galantamine

Better cognitive status at the initiation of ChEI therapy implied a slower rate of progression over time. A higher mean ChEI dose during the study (regardless of drug agent) and a lower level of education were independent predictors of a more positive cognitive long-term outcome in both LOAD models and using the ADAS-Cog model in EOAD. No differences in cognitive trajectories were found among the three ChEI agents in the EOAD or LOAD cohorts. An interaction effect showed that a higher level of education led to increased cognitive impairment over the course of the disease. Female sex, younger age and less-preserved IADL capacity at the start of ChEI treatment were factors that implied a higher rate of cognitive deterioration in LOAD. Interaction effects between age (or sex in the ADAS-Cog mixed-effects model) and cognitive ability at baseline demonstrated that this difference in cognitive performance between age groups (or sex) was more pronounced among the older individuals who were more cognitively impaired. The interaction term of age with ChEI dose was not significant in any of the models. According to the MMSE model, the use of antipsychotics in participants with LOAD implied worse prognosis.

Below, we provide non-linear regression models for calculation of the predicted ADAS-Cog score for a cohort of ChEI-treated patients with EOAD and LOAD, based on the respective baseline score. These equations are intended to predict the scores at subsequent evaluations over a 3-year period. The ADAS-Cog models explained a substantial degree of variance in the dataset; that is, they displayed a good fit (EOAD *R* ^2^ = 0.577, *R* = 0.760, *p* < 0.001; LOAD *R* ^2^ = 0.537, *R* = 0.733, *p* < 0.001).

Predicted ADAS-Cog score in EOAD was calculated as follows:

$$ \widehat{\mathrm{Y}}=0.8227\hbox{--} \left(0.0154\times \mathrm{t}\right)+\left(1.0855\times {\mathrm{x}}_{\mathrm{i}}\right)+\left(0.0178\times {\mathrm{tx}}_{\mathrm{i}}\right)\hbox{--} \left(0.0077\times {{\mathrm{x}}_{\mathrm{i}}}^2\right) $$

Predicted ADAS-Cog score in LOAD was calculated as follows:

$$ \widehat{\mathrm{Y}}=3.1545\hbox{--} \left(0.0456\times \mathrm{t}\right)+\left(0.7455\times {\mathrm{x}}_{\mathrm{i}}\right)+\left(0.0156\times {\mathrm{tx}}_{\mathrm{i}}\right) $$

where *t* is the time in months between baseline and the actual visit and *x*_i_ is the baseline ADAS-Cog score.

## Discussion

In this study performed in routine clinical practice, the 6-month cognitive and global responses to ChEI therapy and the longitudinal outcomes after 3 years were similar between the age-at-onset groups; however, a somewhat faster decline in the EOAD group at some time points was detected when we used the ADAS-Cog scale. Homozygote APOE ε4 carriers were more frequent among the younger patients, but APOE genotype did not significantly affect disease progression in the multivariate models. The EOAD cohort received a higher ChEI dose than the LOAD group over the study period. A higher mean dose of ChEI, better cognitive status at the initiation of treatment and lower level of education were independent protective factors for a more favourable long-term cognitive performance in both groups. Risk factors for worse prognosis in LOAD were female sex, younger age, more impaired IADL capacity and use of antipsychotics.

We defined EOAD as the onset of AD before 65 years of age, which is the definition used most often in earlier studies [21]. Typically, AD has an insidious and gradual onset, and it could sometimes be problematic to distinguish from an age-related deterioration in the beginning of the disease; therefore, the individual’s age at the onset of symptoms might be difficult to estimate accurately [32]. The age cut-off of < 65 years is arbitrary and not based on any biological differences; instead, a social factor, namely the traditional retirement age in many countries, has been used as the dividing line [21, 33]. However, some studies have used other cut-offs for EOAD, such as age at onset < 60 years [7], age at onset ≤ 66 years [33], time of AD diagnosis < 65 years [2, 6], time of AD diagnosis ≤ 65 years [34] and < 79 years at death [5]. This lack of consensus makes comparisons between studies difficult.

The 3-year cognitive outcomes were similar between the onset groups in the present study; however, when using the more complex and sensitive ADAS-Cog scale, a slightly more rapid deterioration in EOAD at the 12-, 18- and 30-month evaluations was found. Inconsistently, in a recent publication derived from a meta-database that included ten AD studies, Schneider et al. [35] observed greater worsening on both ADAS-Cog and MMSE scales over 12–24 months in younger participants, whereas in an earlier study, Kramer-Ginsberg et al. [36] reported no difference between EOAD and LOAD in change in ADAS-Cog + ADAS-Noncog score over the course of up to 2 years. In line with our findings regarding the MMSE scale, a similar worsening regardless of age at onset has been described [19, 37], although other AD studies have suggested a more pronounced decline in MMSE score in younger versus older individuals [16, 34]. In a smaller-sample study (*n* = 42) [37], the mean MMSE changes/year for the antidementia drug-treated patients with EOAD and LOAD were, respectively, 0.82 and 1.0 points, whereas the corresponding rates of progression in the somewhat more cognitively impaired SATS cohort at baseline were slightly faster at 1.1 and 1.4 MMSE points/year. These mixed observations show that the scale used did not provide the sole explanation for these various results, and it is not possible to conclude whether the EOAD group has a different cognitive trajectory than the LOAD cohort. A possibly more rapid disease course in younger participants and unequal age distributions across cohorts included in clinical trials may affect the results of these studies.

In the present study, males with LOAD exhibited more positive longitudinal cognitive abilities than females. Male sex has been related to a slower progression rate in some multivariate studies [38, 39] but not all [40]. However, these reports did not address the potential impact of ChEI therapy. A more positive short-term cognitive response to ChEIs among males was demonstrated by our group [13] and also by a 3-month study of tacrine and galantamine [41]. One explanation for these sex differences might be the role of sex hormones in AD [42]. Another theory is that males have larger cerebral hemispheres than women even after controlling for body size [43]. In addition, the association between AD pathology and dementia was reported as more pronounced in women than in men [44]. These findings could indicate that men withstand AD pathology better and that the female brain is more vulnerable, which might explain the more favourable cognitive outcome over time and the better response to ChEIs shown among men. However, the possible impact of sex regarding differences in the prognosis of AD and the efficacy of ChEI treatment requires further investigation.

The younger cohort in this study exhibited a significantly higher education level, longer duration of AD and better functional performance but similar cognitive status at baseline. These characteristics have been described in other reports on EOAD [33]. More years of education were related to a faster deterioration in cognitive abilities in both our age-at-onset groups. This observation supports the cognitive reserve hypothesis, according to which more highly educated people are expected to have a more advanced disease at the time of AD diagnosis [45]. Moreover, neuropathological studies detected a higher burden of AD pathology and larger synapse loss in younger than in older patients [4]. In the present study, the somewhat more rapid decline in individuals with EOAD measured by ADAS-Cog might reflect their higher education and more pronounced cognitive reserve. The rate of disease progression has been suggested to increase in the moderate to severe stages of AD using the traditional cognitive assessment scales [40]. Furthermore, older age in LOAD was associated with better long-term cognitive outcome in this study. A reduced cognitive reserve capacity among the oldest participants could lead to earlier detection of the disease, diagnosis of AD, and start of ChEIs at an earlier stage, which could improve efficacy. Our finding that a longer illness duration before AD diagnosis was demonstrated in our EOAD cohort also supports this explanation. Taken together, a more advanced disease with greater AD pathology at baseline and thus later initiation of ChEI therapy might occur in the younger SATS group.

In the present study, a higher frequency of homozygote APOE ε4 carriers was observed in the EOAD cohort; however, APOE genotype did not affect the cognitive trajectories in the multivariate models for either the younger or the older patients in the SATS. Earlier studies of the relationship of the ε4 allele with rate of progression in AD were inconsistent [46, 47]. One study showed that APOE non-ε4 carriers with EOAD had a faster cognitive deterioration than non-ε4 carriers with LOAD; however, the level of education of the groups was not mentioned [34]. Previously, we reported that ε4 carriers were younger at the start of ChEI treatment and had more years of education than the non-ε4 carriers [13]. Younger individuals may have more hereditary and aggressive forms of AD [48]. Different patient characteristics among studies, such as education level and thus cognitive reserve, as well as genetic predispositions between the age-at-onset groups might lead to various outcomes.

Use of antipsychotics and lower IADL performance were risk factors for a more pronounced cognitive decline in participants with LOAD in this study. Hearing and visual impairment among older persons with dementia could lead to more delusions and visual hallucinations, respectively [49]. A review of psychosis in AD indicated that the association between age at onset and psychotic symptoms was inconsistent among studies. However, psychosis led to a faster cognitive worsening in all included studies in which researchers investigated this issue [50]. In addition, antipsychotic therapy in AD was related to significant cognitive progression over time compared with placebo [51]. These findings suggest that individuals with psychotic symptoms consist of a subset with a more aggressive course of AD and worse prognosis. The LOAD cohort had more functional deficits than the EOAD group in this study. Our group and others have reported that patients with AD with rapid IADL deterioration exhibit significantly lower cognitive status at the initial evaluation [22, 52]. Moreover, a moderate linear association between cognition and function has been demonstrated in AD [53], which suggests that these aspects should be interpreted simultaneously in multivariate models because a change in one capacity could influence a change in another. This interaction effect might have implications for the results reported in pharmaceutical trials of new AD drug agents.

The present observational, longitudinal study is the first to show the potential impact of ChEI treatment among participants with EOAD and LOAD separately. The clinical value of ChEIs is still controversial among physicians. The authors of a recent publication from the Swedish Dementia Registry stated that the prevalence of ChEI therapy was significantly higher among the EOAD group than the LOAD cohort (88% versus 75%); that is, one of four older individuals diagnosed with AD did not receive ChEIs [2]. In our study, no differences in effectiveness among the three drug agents were observed. Higher doses of ChEIs were related to a more positive cognitive outcome in both age-at-onset groups; however, the patients with LOAD received a lower dose over time than those with EOAD. It is not known whether this finding depended on the older person’s actual lower tolerability or the physician’s opinion that an older individual might not tolerate a higher ChEI dose. The association between a higher dose of ChEI and slower disease progression has been reported among the total number of SATS participants with mild to moderate AD [13], as well as in a meta-analysis of randomised trials [54].

The strengths of the SATS are the prospective, well-structured assessments every 6 months over 3 years after the initiation of ChEIs in a large cohort of continuously treated ordinary patients with AD with co-morbidities and concomitant medications from memory clinics across Sweden. The 3-year completion rates of 40% and 36% obtained for the EOAD and LOAD participants from clinical practice, respectively, were high compared with other AD extension or naturalistic studies (20–39%) [23]. The large drop-out rate in all long-term AD studies may contribute to a more favourable outcome for the remaining patients, assuming that they benefit more from ChEI therapy. Our results show that the completers in both age-at-onset groups received a higher mean dose of ChEI during the study, suggesting a better tolerance of the treatment. In the mixed-effects models, the outcomes of the drop-outs were also included during their time of participation. The lower cognitive and global abilities at baseline observed for the non-completers with LOAD may contribute to trajectories that are more positive for the older individuals remaining in the SATS over time. Patients with LOAD and psychotic symptoms exhibited a higher risk of discontinuing the study. However, the drop-outs were similar to the completers regarding the other characteristics. One limitation is that the SATS was not placebo-controlled owing to ethical concerns or randomised with respect to ChEI drug agent, similar to other longitudinal, observational AD studies. Specialists in dementia disorders decided on the type of ChEI and dose for each participant, in agreement with the standards used in a routine clinical setting. Another shortcoming of this study, as in previous publications on age at onset [55], is that the physician’s estimation of onset of AD symptoms relied on information and retrospective observations of the caregiver and their attention.

Few long-term studies have analysed the relationships between EOAD and LOAD, APOE genotype, level of education and concomitant medications. Moreover, earlier findings are not consistent regarding the effect of, for example, age at onset and APOE ε4 carrier status on disease prognosis; thus, additional studies are warranted. The short-term response to ChEIs and the effect over a longer time in different age groups, as well as possible predictors that might alter the outcome, have not been investigated previously.

## Conclusions

A comparison of various aspects of disease progression between EOAD and LOAD was performed in this observational study. After 3 years, the cognitive and global rates of decline were similar between the age-at-onset groups; however, the more sensitive ADAS-Cog scale tended to exhibit a faster worsening among the younger individuals over this period. This information is necessary for the interpretation of results from clinical trials to evaluate the effectiveness of and provide realistic expectations for new potentially disease-modifying therapies (as add-ons to ChEIs) directed at AD cohorts of various ages. In addition, male sex, better IADL performance and no use of antipsychotics in the LOAD group, as well as fewer years of education in both groups, were protective factors of a more positive longitudinal cognitive outcome. Although the patients with EOAD included a larger proportion of carriers of two APOE ε4 alleles, this observation did not influence progression rate using multivariate models. The socio-demographic and clinical composition of an AD cohort under study may be one explanation for the heterogeneity of results presented in a number of reports. A higher mean dose of ChEI (regardless of drug agent) was associated with slower cognitive decline in both onset groups, but the older participants received a lower dose during most of the 3-year study period. This finding stresses the importance for clinicians to optimise the ChEI dose in AD, regardless of the individual’s age at onset, to improve treatment effectiveness. In summary, our results suggest that EOAD and LOAD are not separate entities. The younger patients’ longer time to AD diagnosis, higher level of education and thus cognitive reserve capacity might explain most of the differences detected between the groups.

## Abbreviations

AD: Alzheimer’s disease
ADAS-Cog: Alzheimer’s Disease Assessment Scale–Cognitive subscale
ADL: Activities of daily living
APOE: Apolipoprotein E
ChEI: Cholinesterase inhibitor
CIBIC: Clinician Interview-Based Impression of Change
EOAD: Early-onset Alzheimer’s disease
IADL: Instrumental Activities of Daily Living Scale
LOAD: Late-onset Alzheimer’s disease
MMSE: Mini Mental State Examination
NSAID: Non-steroidal anti-inflammatory drug
PSMS: Physical Self-Maintenance Scale
SATS: Swedish Alzheimer Treatment Study
